# Hemorrhagic shock due to colonic arteriovenous malformation in late pregnancy: a case report

**DOI:** 10.1186/s12245-022-00424-6

**Published:** 2022-05-17

**Authors:** Toshinao Suzuki, Satoru Murata

**Affiliations:** Interventional Radiology Center, Teikyo University Chiba Medical Center, 3426-3 Anesaki, Ichihara, Chiba, 299-0111 Japan

**Keywords:** Arteriovenous malformations, Pregnancy, Shock, Gastrointestinal hemorrhage, Therapeutic embolization, Angiography

## Abstract

**Background:**

Intestinal arteriovenous malformations are difficult to detect because they often present asymptomatically. However, pregnancy increases the hemorrhagic risk of intestinal arteriovenous malformations. This can lead to massive bleeding and hemodynamic instability, threatening the lives of both the mother and fetus. We describe a life-threatening case of hemorrhagic shock due to a colonic intestinal arteriovenous malformation during late pregnancy that was successfully treated through endovascular management.

**Case presentation:**

A 36-year-old gravida 1, para 1 woman at 35 weeks’ gestation presented with hemodynamic instability and painless hematochezia. The patient had hemorrhagic shock and required massive transfusion. A colonoscopy failed to secure a visual field due to bloody fluid, and endoscopic hemostasis was difficult. Before the bleeding could be controlled, the condition of the fetus continued to deteriorate, showing bradycardia dysrhythmia. Therefore, an emergency cesarean section was performed, which was successful. However, the bleeding did not subside, with the patient’s hemodynamic instability and hematochezia persisting. An angiogram revealed an ascending colonic intestinal arteriovenous malformation, with extravasation of the contrast medium from a branch of the ileocolic artery. Localized blood flow control and hemodynamic stability were achieved via angioembolization. The patient had an uneventful postoperative recovery and was discharged on postoperative day 12. The newborn was admitted to the neonatal intensive care unit. She successfully recovered and was discharged when she was 22 days old.

**Conclusions:**

We reported a case of colonic intestinal arteriovenous malformation resulting in hemodynamic instability due to hematochezia during late pregnancy, which was successfully treated via angioembolization. Intestinal arteriovenous malformation should be considered as a differential diagnosis in pregnant patients with hemodynamic instability and hematochezia.

## Background

Intestinal arteriovenous malformations (AVMs) often cause intestinal bleeding. However, most cases have remained undiagnosed due to the absence of clinical symptoms. Pregnancy rapidly increases the size, blood flow, and risk of bleeding of AVMs [1–3]. Therefore, bleeding secondary to an intestinal AVM may suddenly occur in pregnant women. Nevertheless, the prevalence of intestinal AVMs in pregnant women is unknown. Intestinal AVM causes massive hemorrhage and hemodynamic instability, endangering the lives of both the mother and fetus [4, 5]. We describe a life-threatening case of hemorrhagic shock due to a colonic AVM during late pregnancy that was successfully treated with endovascular management.

## Case presentation

A 36-year-old, gravida 1, para 1 woman, who was 35 weeks pregnant, was admitted to another hospital due to syncope and massive hematochezia (estimated volume, 1 L). She was taking iron pills for iron deficiency anemia associated with pregnancy (hemoglobin value 1 month ago was 7.4 mg/dL). Even though her stools were usually black, she passed fresh bloody stools without abdominal pain. She had no family history of AVM or bleeding disorder.

On arrival at the previous hospital, the patient was in hemorrhagic shock, with a blood pressure of 60/38 mmHg, pulse rate of 92 beats/min, oxygen saturation of 100% with an oxygen mask inhalation of 10 L/min, and body temperature was 37°C.

A peripheral venous line was inserted, and crystalloid and blood transfusion were started. The patient was transferred to the emergency department of our hospital while being given rapid blood transfusions due to hemodynamic instability, requiring intervention for hemorrhagic shock. Her medical history was unremarkable, and she did not receive anticoagulant or antiplatelet therapy.

**Fig. 1:**
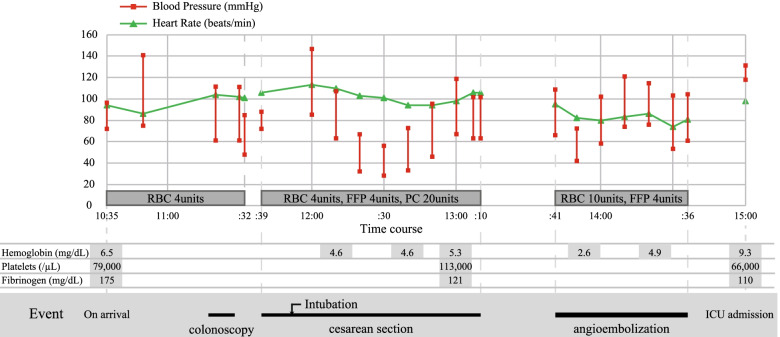
Clinical vital signs chart. RBC; packed red blood cells, FFP; fresh frozen plasma, PC; platelet transfusion, ICU; intensive care unit

On arrival, her blood pressure was 98/72 mmHg, pulse rate 97 beats/min, and oxygen saturation 100% with an oxygen mask inhalation of 10 L/min (Fig. 1).

Physical examination revealed fresh blood in the rectum. Laboratory tests revealed a hemoglobin of 6.5 g/dL, and coagulopathy was confirmed (fibrinogen, 175 mg/dL; platelets, 79,000/μL; prothrombin time, 13.0 s; activated partial thromboplastin time, 35.4 s; and anti-thrombin 3, 37%). She had a white blood cell count of 9.2 × 10^9^/L and a C-reactive protein level of < 0.3 mg/dL (undetectable).

**Fig. 2:**
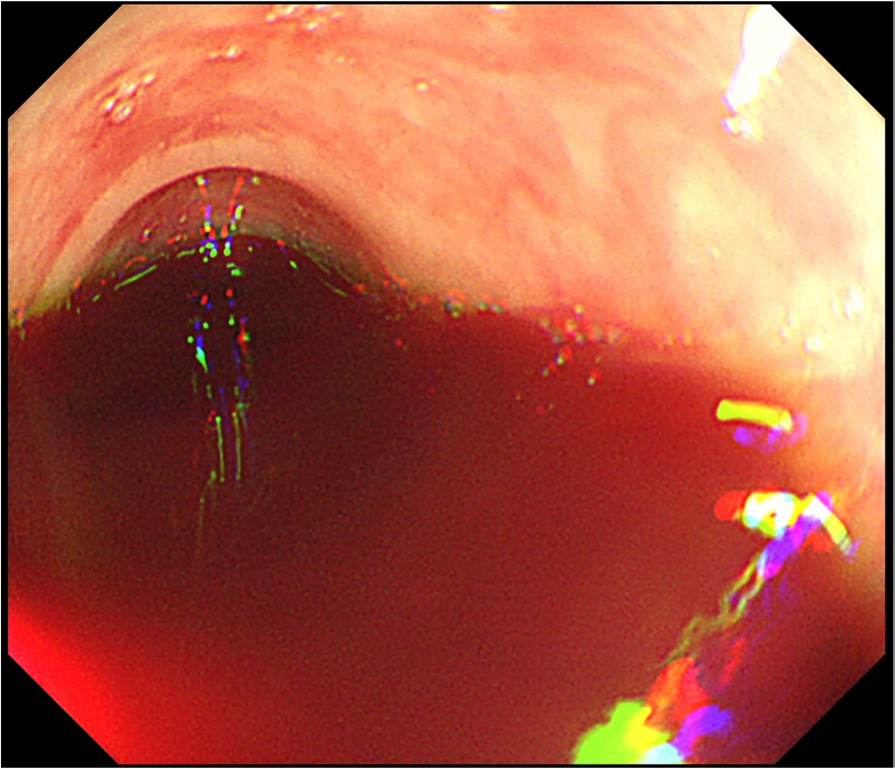
Colonoscopic images of a blood-filled colon. It was difficult to secure the visual field because of the large amount of blood

An emergency cesarean section was deemed necessary. Before the cesarean section, a colonoscopy was performed to identify the bleeding source in the rectum. Bleeding from the rectum was easily controllable. However, the colonoscopy failed to secure a visual field because of the bloody fluid (Fig. 2).

**Fig. 3:**
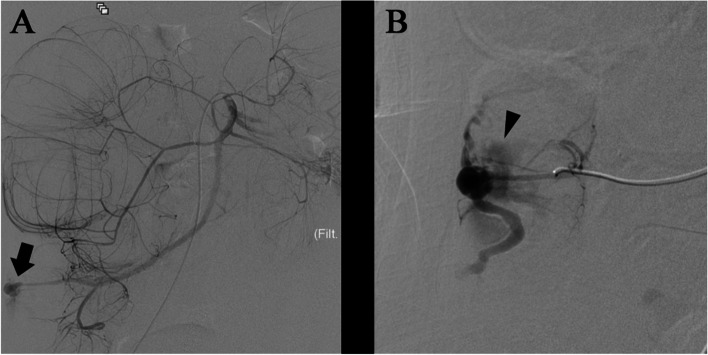
Intraoperative angiography before embolization. **A** Superior mesenteric artery angiography shows contrast extravasation (arrow) in the distal ileocolic artery. **B** Selective ileocolic artery angiography with microcatheter revealed contrast extravasation to the ascending colon lumen (arrowhead) and abnormally dilated vessels

Immediately before entering the operating room, the patient exhibited a decreased level of consciousness, worsening blood pressure, and fetal bradycardia dysrhythmia (heart rate 60 bpm). An emergency cesarean section was then successfully performed (blood loss in the surgical field: 639 mL). The newborn had a birth weight of 2550 g, and the Apgar scores were 1 and 2 at 1 and 5 min, respectively. The patient was transferred to the angiography room under general anesthesia due to persistent hemodynamic instability and hematochezia. A superior mesenteric angiogram revealed an ascending colonic AVM with extravasation of the contrast medium from a branch of the ileocolic artery (Fig. 3A, B).

**Fig. 4:**
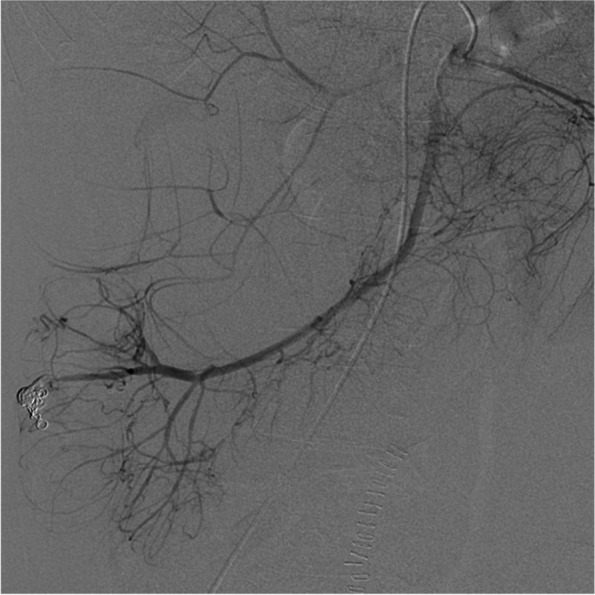
Superior mesenteric artery angiography after embolization. Embolization of the distal ileocolic artery was successfully performed using coils and N-butyl cyanoacrylate, and no contrast leakage was observed

Coils were placed as scaffolds to decrease the blood flow. An additional 33% N-butyl cyanoacrylate and iodized oil mixture were carefully administered to fill the coils. Localized blood flow control and hemodynamic stability were achieved via embolization. Postoperative contrast leakage was not observed (Fig. 4).

When entering the intensive care unit, laboratory tests revealed a hemoglobin of 9.3 g/dL, and coagulopathy was confirmed (fibrinogen, 110 mg/dL; platelets, 66,000/μL; prothrombin time, 15.5 s; activated partial thromboplastin time, 51.9 s) (Fig. 1). The patient required 20 units of packed red blood cells, 16 units of fresh frozen plasma, and 30 units of platelet transfusion throughout the resuscitation while using vital signs and blood tests as indicators. The patient had an uneventful medical recovery and was discharged on postoperative day 12. Six months after her discharge, elective colonoscopy showed no abnormal colonic findings, including in the ascending colon, where the AVM was previously located. The brain magnetic resonance imaging was also unremarkable. The patient remained asymptomatic. The newborn was admitted to the neonatal intensive care unit. She successfully recovered and was discharged when she was 22 days old.

## Discussion and conclusions

This report documented the successful treatment of a colonic AVM with hemorrhagic shock during late pregnancy using endovascular embolization. AVMs are abnormal and directly connect arteries and veins without intermediary capillary beds [4, 6]. They typically occur in the head and neck. In the gastrointestinal tract, they cause acute and chronic bleeding, which can be fatal [4]. Intestinal AVM should be considered a differential diagnosis in pregnant patients with hemodynamic instability and hematochezia. Furthermore, endovascular management is a less invasive option for treating hemorrhage secondary to intestinal AVM.

Hemodynamic instability was reportedly exacerbated by hemorrhage secondary to intestinal AVM [7]. However, there are no reports of intestinal AVM causing hemodynamic instability in pregnant women. To the best of our knowledge, this is the first report of antepartum hemorrhage due to colonic AVM.

In this case, an unidentified AVM can induce hemorrhage due to pregnancy, resulting in hemodynamic instability. The most common chief concern in patients with colonic AVM is hematochezia (56–60%) [6, 8]. However, hemorrhage in AVM is characterized by the absence of abdominal pain [9]. In 25% of AVM cases, the patients are asymptomatic and diagnosed via colonoscopy screening [6, 8]. Most patients were unaware of the presence of intestinal AVMs. Furthermore, pregnancy increases the risk of AVM expansion and hemorrhage [1, 2]. Pregnancy stimulates a rapid increase in size and blood flow of the AVM [3]. In cerebral AVMs, the bleeding risk in pregnant women was reportedly 27%, which was four times higher than that in the general population [1, 10]. Data regarding intestinal AVMs are insufficient. However, hemorrhage secondary to intestinal AVM can be severe and life-threatening for both the mother and fetus.

Hemorrhage due to AVM has life-threatening complications, such as maternal shock, increased risk of premature delivery, fetal hypoxia, and sudden fetal death [5]. In this case, the patient developed hemorrhagic shock. Intestinal AVM was diagnosed and treated with endovascular management. An emergency cesarean section was necessary to save the fetus. Immediate angiography is warranted in patients with hemorrhagic shock who experience acute overt intestinal hemorrhage; computed tomography is not always necessary [11]. Intestinal AVM, in this case, was successfully diagnosed via angiography, and hemorrhage control was achieved via minimally invasive embolization [4]. Although intestinal AVMs are difficult to recognize, early identification and intervention are necessary because the condition worsens throughout pregnancy, threatening both the mother and fetus.

We reported a case of colonic AVM, which resulted in hemodynamic instability due to hematochezia during late pregnancy. The patient was successfully treated with angioembolization. A differential diagnosis of intestinal AVMs should be considered in pregnant patients with hemodynamic instability and hematochezia.

## Data Availability

Data sharing is not applicable to this article as no datasets were generated or analyzed during the current study.

## Abbreviations

AVM: Arteriovenous malformation

## References

[1] Lv X, Liu P, Li Y (2015). The clinical characteristics and treatment of cerebral AVM in pregnancy. Neuroradiol J..

[2] Liu AS, Mulliken JB, Zurakowski D, Fishman SJ, Greene AK (2010). Extracranial arteriovenous malformations: natural progression and recurrence after treatment. Plast Reconstr Surg..

[3] Manzocchi Besson S, Jastrow Meyer N, Bounameaux H, La Scala GC, Calza AM, Yilmaz H (2019). Multiple arteriovenous malformations caused by RASA1 gene mutation presenting during pregnancy - a case report and review of the literature. Vasa..

[4] So M, Itatani Y, Obama K, Tsunoda S, Hisamori S, Hashimoto K (2018). Laparoscopic resection of idiopathic jejunal arteriovenous malformation after metallic coil embolization. Surg Case Rep..

[5] Walfish M, Neuman A, Wlody D (2009). Maternal haemorrhage. Br J Anaesth..

[6] Lee HH, Kwon HM, Gil S, Kim YS, Cho M, Seo KJ (2017). Endoscopic resection of asymptomatic, colonic, polypoid arteriovenous malformations: two case reports and a literature review. Saudi J Gastroenterol..

[7] Mazahreh TS, Aleshawi AJ, Alorjani MS, Elayyan R, Al-Zoubi NA (2019). Arteriovenous malformations within jejunal diverticulosis: case report and literature review. BMC Surg..

[8] Rzepczynski A, Kramer J, Jakate S, Cheng L, Singh A (2019). Colonic polypoid arteriovenous malformation causing symptomatic anemia. ACG Case Rep J..

[9] Wilkins T, Baird C, Pearson AN, Schade RR (2009). Diverticular bleeding. Am Fam Physcian.

[10] Robinson JL, Hall CS, Sedzimir CB (1974). Arteriovenous malformations, aneurysms, and pregnancy. J Neurosurg..

[11] Gerson LB, Fidler JL, Cave DR, Leighton JA (2015). ACG clinical guideline: diagnosis and management of small bowel bleeding. Am J Gastroenterol..

